# Plasmatic Dimethylarginines in Dogs With Myxomatous Mitral Valve Disease

**DOI:** 10.3389/fvets.2021.738898

**Published:** 2021-09-16

**Authors:** Carlotta Valente, Carlo Guglielmini, Marco Baron Toaldo, Giovanni Romito, Carlo Artusi, Laura Brugnolo, Barbara Contiero, Helen Poser

**Affiliations:** ^1^Department of Animal Medicine, Production and Health, University of Padua, Padua, Italy; ^2^Department of Veterinary Medical Sciences, Alma Mater Studiorum–University of Bologna, Bologna, Italy; ^3^Department of Laboratory Medicine, University-Hospital of Padua, Padua, Italy

**Keywords:** ADMA, canine, heart failure, SDMA, arginine, dimethylarginines

## Abstract

Plasmatic dimethylarginines, asymmetric dimethylarginine (ADMA) and symmetric dimethylarginine (SDMA) are considered biomarkers of endothelial and renal dysfunction, respectively, in humans. We hypothesize that plasmatic concentration of dimethylarginines in dogs with myxomatous mitral valve disease (MMVD) is influenced by heart disease stage. Eighty-five client-owned dogs with MMVD, including 39, 19, and 27 dogs in ACVIM stages B1, B2, and C+D, respectively, and a control group of 11 clinically healthy dogs were enrolled. A prospective, multicentric, case-control study was performed. Each dog underwent a complete clinical examination, arterial blood pressure measurement, thoracic radiography, six-lead standard electrocardiogram, transthoracic echocardiography, CBC, biochemical profile, and urinalysis. Plasmatic concentration of dimethylarginines was determined through high-performance liquid chromatography coupled with tandem mass spectrometry. Median ADMA was significantly increased in dogs of group C+D (2.5 μmol/L [2.1–3.0]) compared to those of group B1 (1.8 μmol/L [1.6–2.3]; *p* < 0.001) and healthy dogs (1.9 μmol/L [1.7–2.3]; *p* = 0.02). Median SDMA was significantly increased in dogs of group C+D (0.7 μmol/L [0.5–0.9]) compared to those of groups B1 (0.4 μmol/L [0.3–0.5]; *p* < 0.001), B2 (0.4 μmol/L [0.3–0.6]; *p* < 0.01), and the control group (0.4 μmol/L [0.35–0.45]; *p* = 0.001). In the final multivariable analysis, ADMA and SDMA were significantly associated with left atrium to aorta ratio (*p* < 0.001), and creatinine (*p* < 0.001), respectively. Increased plasmatic concentrations of dimethylarginines suggest a possible role as biomarkers of disease severity in dogs with decompensated MMVD.

## Introduction

Dimethylarginines (DMAs) are biological products derived by the methylation process of L-arginine. Among them, asymmetric dimethylarginine (ADMA) and its stereoisomer, symmetric dimethylarginine (SDMA), are the most frequently investigated as circulating biomarkers.

Despite their common origin, ADMA and SDMA have different clearance pathways, and consequently, are involved in different pathophysiological processes. Asymmetric dimethylarginine is transported to the kidneys, brain, and liver where it is mainly metabolized by dimethylarginine dimethylaminohydrolase (1) and only a minimal portion is excreted by the kidneys, whereas SDMA is almost completely eliminated through renal excretion (2).

Asymmetric dimethylarginine acts as an endogenous nitric oxide synthase (NOS) inhibitor, impairing nitric oxide (NO) production (3), which is involved in the homeostasis of vascular tone and blood pressure (4). The L-arginine/ADMA ratio has been studied as an index of NO bioavailability and this ratio seems to be reduced in humans with endothelial dysfunction (5). On the contrary, SDMA is not a direct inhibitor of NO production, but competing with L-arginine cellular uptake, it contributes to reducing NOS activity. Symmetric dimethylarginine is mainly considered a marker of renal damage (6), whereas ADMA is considered a biomarker of endothelial dysfunction (5, 7–10). High levels of both ADMA and SDMA are considered risk factors for cardiovascular and renal morbidity and mortality in humans (11).

Myxomatous mitral valve disease (MMVD) is the most common acquired cardiac disease in dogs and can eventually lead to congestive heart failure (CHF). The American College of Veterinary Internal Medicine (ACVIM) has classified MMVD into four main stages of severity based on clinical, radiographic and echocardiographic parameters (12). Symmetric dimethylarginine has been validated both in dogs and cats as a reliable, early and specific biomarker of renal damage, but little information is available about the role of DMAs in canine cardiovascular diseases (13). Increased SDMA concentration has been described in dogs with MMVD according to disease severity, leading to the hypothesis that impaired cardiac function might consequently alter renal activity (14), whereas ADMA has previously been studied in healthy dogs and in dogs with MMVD in the preclinical stages of disease (15, 16). Understanding the role of DMAs in dogs with cardiac disease can be helpful in recognizing potential risk factors. Furthermore, evaluation of DMAs can be an adjunctive and non-invasive diagnostic tool for the identification of early impaired cardiac and/or renal function in these animals. The aim of this study was to evaluate if plasmatic DMA concentrations in dogs with MMVD are influenced by heart disease stage. We hypothesized that plasmatic levels of ADMA and SDMA increase according to the progression of MMVD and that the L-arginine/ADMA ratio decreases in the most advanced stage of disease.

## Materials and Methods

### Study Design and Animals

The protocol of this prospective, multicentric, case-control study was approved by the University of Padua Animal Welfare Ethics Committee (Authorization number 26/2017).

Dogs were prospectively enrolled from September 2017 to September 2019 at the Veterinary Teaching Hospital of the University of Padua and the University of Bologna. All animals were recruited among external or internal cardiological referral cases or dogs evaluated for a health status check-up. After written informed consent was signed by the dog's owner, each animal underwent a complete physical examination, indirect measurement of arterial systemic pressure through high definition oscillometry (VET HDO Monitor MD PRO-it), six-lead standard electrocardiography (Cardioline Touch ECG, Cardioline s.p.a., Trento Italy), survey thoracic radiography, echocardiographic examination, including real-time 2D, M-mode and echo-Doppler evaluation, and blood and urine sampling for CBC and serum biochemistry profile. Urinalysis, including determination of urinary protein to creatinine ratio and urine specific gravity (USG) was also performed.

Small- and medium-sized dogs with body weight (BW) <20 kg, aged over seven years, with a diagnosis of MMVD, and absence of other heart or systemic diseases were enrolled. The severity of MMVD was defined according to the ACVIM guidelines (12). In particular, asymptomatic dogs without radiographic or echocardiographic evidence of cardiac remodeling were considered as stage B1; dogs were considered as stage B2 if diagnostic imaging evidenced cardiac remodeling (i.e., cardiomegaly with left atrial and ventricular enlargement). Symptomatic dogs in which at least one episode of pulmonary edema and/or pleural effusion due to CHF had occurred were considered as stage C; symptomatic dogs with end-stage disease and refractory to standard cardiac treatment were considered as stage D. Due to the few animals classified in stage D of MMVD, these dogs were merged with those in stage C for statistical evaluation (group C+D) (12).

Dogs referred to the cardiology units for general screening or pre-anesthetic evaluation for surgery (i.e., orthopedic or neutering surgery), without any clinical, echocardiographic, and laboratory signs of cardiovascular or systemic diseases were enrolled as the control group.

### Cardiac Imaging

Right lateral and dorsoventral or ventrodorsal radiographic views of the thorax were obtained and the vertebral heart score was calculated from the lateral view as previously described (17).

The transthoracic echocardiographic examination was performed by experienced operators (CG, HP, MBT) in awake animals from the right and left parasternal windows using standard views (18). Commercially available ultrasound units (CX50, Philips, Eindhoven, Netherlands and iE33, Philips Healthcare, Monza, Italy), equipped with multi-frequency phased array transducers, were employed with continuous ECG monitoring.

Left ventricle measurements, including the internal diameter of the left ventricle in diastole and systole (LVIDd and LVIDs, respectively) were obtained from the right parasternal window, short-axis view at the level of *chordae tendinae* using the 2D guided M-mode. The internal diameters of the left ventricle were indexed to the BW using the formula: normalized LVIDd (LVIDd-N) = LVIDd/[BW^0.294^] and normalized LVIDs (LVIDs-N) = LVIDs/[BW^0.315^] (19). Left ventricular enlargement was considered for values of LVIDd-N ≥ 1.7. The left atrium (LA) and aortic diameter (Ao) were measured from the right parasternal window using a short-axis view at the level of the aortic root with the 2D method and measurements taken at early diastole. From these variables, the left atrium to aortic root ratio (LA/Ao) was then calculated and LA enlargement was considered for a LA/Ao ≥ 1.6 (20–22). From the left parasternal window, apical four-chamber view, early and late diastolic trans-mitral (MV E and MV A, respectively) and trans-tricuspid (TV E and TV A, respectively) peak diastolic velocities were obtained using pulsed-wave Doppler, placing the sample volume on the ventricular side at the tip of the mitral or tricuspid valve's leaflets, respectively. When systolic regurgitant blood flow was observed on color-flow mapping of the tricuspid valve, tricuspid regurgitation (TR) peak velocity was recorded, trying to adjust the interrogation beam as parallel as possible to the regurgitant jet. A value of TR peak velocity ≥ to 3.4 m/s (corresponding to a systolic pressure gradient ≥ 46 mmHg using the modified Bernoulli equation) was used to diagnose pulmonary hypertension in dogs without obstruction of the right ventricular outflow (23). Pulsed-wave tissue Doppler imaging was studied from the left apical four-chamber view, with the sample gate positioned on the lateral and septal mitral annulus, and, after optimizing the alignment of the ultrasound with right ventricle free wall, on the lateral tricuspid annulus. For each position, we considered measurements of the peak velocity of early diastolic lateral and septal mitral annular motion (LatMV E' and SepMV E', respectively) and of the peak velocity of early diastolic lateral tricuspid annular motion (TV E').

Each echocardiographic variable was measured in triplicate, using the average value for the subsequent statistical analysis.

### Laboratory Analysis

Complete blood count and serum biochemical analyses were performed at the laboratories of Veterinary Teaching Hospital of the University of Padua and the University of Bologna within 24 h of blood collection, and urinalysis within 4 h of urine sampling, with dogs fasted for 12 h beforehand. The hematologic parameters were measured using automated analyzers (Advia 120, Hematology System, Siemens, Italy; Advia 2120, Siemens Healthcare Diagnostics, Erlangen, Germany). The biochemical variables and urinary protein to creatinine ratio were evaluated using other automated analyzers (BT1500, Biotecnica, Roma, Italy; AU 480, Beckman Coulter-Olympus, Brea, California, USA). Normal and pathological internal quality controls were performed daily for both analyzers, whereas external control quality was performed once a month.

Urine physical and chemical examinations were performed using a commercial urinalysis stick (DIRUI A10). Then, the USG was determined using a refractometer. Finally, after centrifugation, the urinary sediment was examined.

Blood used for hematological analysis was collected in vacutainer tubes containing K3-EDTA. They were centrifuged at 3,000 rpm for 10 min, then about 0.5 ml of plasma was separated and stored at −20 °C for successive DMA analyses. Plasmatic levels of ADMA, SDMA, and L-arginine were measured simultaneously at the laboratory of University-Hospital of Padua using high-performance liquid chromatography coupled with tandem mass spectrometry (Agilent 1200 Series LC system coupled with an Agilent 6430 triple quadrupole, Palo Alto, CA, USA), as previously described (24, 25). Quantification of N-terminal propeptide of B-type natriuretic peptide (NT-proBNP) was obtained from plasmatic samples by a referring laboratory (IDEXX Laboratories, Leipzig, Germany), through the Cardiopet® NT-proBNP method.

### Statistical Analysis

A preliminary analysis was conducted to calculate the minimum sample size necessary to obtain significant results. In particular, the determination of sample size was based on the studies of Nabity et al. (13) and Pedersen et al. (16). Considering a power of the statistical test equal to 80% and a type I error equal to 5%, 11 dogs for each group were adequate to check a standardized difference of 1.27 and 1.30 for SDMA and ADMA, respectively.

The statistical analysis was performed with commercially available statistical software (SAS®, 9.2 SAS Institute Inc; XLStat Addinsoft (2021) – XLSTAT statistical and data analysis solution, Paris, France; and MedCalc Statistical Software, version 19.3.1 – MedCalc Software Ltd., Ostend, Belgium).

Normal distribution of data was assessed using the Shapiro–Wilk's test. As the variables were not normally distributed, the differences among groups were analyzed using a non-parametric approach (Kruskal–Wallis test). *Post hoc* pairwise comparisons among levels were tested using the Steel–Dwass–Critchlow–Fligner procedure.

Differences in categorical variables, expressed as count data, were tested using the Chi-squared test. The association between clinical, laboratory, and echocardiographic variables with DMAs and L-arginine/ADMA were studied using the Spearman's rank correlation index. The variables showing the most significant correlations were successively evaluated in a multivariable model, to assess their effect on DMAs and L-arginine/ADMA.

Data are expressed as median and interquartile range. An overall *p* < 0.05 was considered as statistically significant, whereas a *p* < 0.001 was set for Spearman's correlations.

## Results

### Study Population

Ninety-six dogs met the inclusion criteria, including 11 (11.5%) control dogs and 85 (88.5%) dogs with MMVD at different stages. Of all enrolled dogs, 49 (51%) were mongrels, followed by nine (9.3%) Cavalier King Charles Spaniels, four (4.1%) Jack Russell Terriers, and four (4.1%) Miniature Pinschers. All less represented breeds are reported in Table 1. Forty-four (45.8%) dogs were females and 52 (54.2%) males. The median (interquartile range) age was 11 years (9–14 years) and the median BW was 9.1 kg (6.7–12.1 kg). Dogs in the control group were younger compared to those of group C+D (*p* = 0.014).

**Table 1:** Clinical parameters of the 96 dogs included in the study.

**Parameter**	**Control**	**B1**	**B2**	**C+D**	**Overall *p*-value**
N. of dogs (%)	11 (11)	39 (41)	19 (20)	27 (28)	
Age (Y)	9 (7–12)	11 (8–13)	12 (11–14)	12 (10–14) ^*^	0.01
BW (Kg)	9 (7.2–17.5)	10.5 (8–13.6)	7.3 (5.4–10.9)	9 (7.5–13.8)	0.23
Sex M/F	3/8	22/17	12/7	15/12	0.27
Breed (N.)	Mongrel (4), Miniature Pinscher (2), Australian Shepherd, Beagle, WHWT, English Bulldog, Pug (1)	Mongrel (18), CKCS (6), Dachshund (2), Jack Russell Terrier (2) English Setter (2), Maltese, Fox Terrier, Russian Toy, Yorkshire Terrier, Miniature Pinscher, Miniature Schnauzer, Spitz, Cocker Spaniel, Pug (1)	Mongrel (10), CKCS (3), Pomeranian Fox, Miniature Schnauzer, Miniature Pinscher, Bolognese, Shih-Tzu, Greyhound (1)	Mongrel (17), Jack Russell Terrier (2), Yorkshire Terrier, Beagle, Bolognese, Border Collie, Shih-Tzu, Maltese, Epagneul Breton, English Setter (1)	0.41
PH (%)	0 (0)	1 (2.5)	4 (21)	6 (22)	0.05
Cardiovascular treatment, N. (%)	0 (0)	8 (20)	12 (63)^**^	26 (96)^**^, ^***^	<0.001
Furosemide, N. (%) [doses range] mg/kg	0 (0)	5 (13) [0.5–5.0]	4 (21) [0.6–4.0]	23 (88) [0.6–9.0]	
Benazepril, N. (%) [doses range] mg/kg	0 (0)	7 (18) [0.2–0.8]	5 (26) [0.16–0.5]	20 (74) [0.25–1.1]	
Spironolactone, N. (%) [doses range] mg/kg	0 (0)	1 (3) [2.3]	0 (0)	6 (22) [1.0–3.0]	
Pimobendan, N. (%) [doses range] mg/kg	0 (0)	2 (5) [0.5–0.6]	9 (47) [0.4–0.8]	19 (70) [0.1–0.8]	

*Data are presented as median and interquartile range.*

*N., number of dogs; BW, bodyweight; PH, pulmonary hypertension; H, clinically healthy dogs; B1, dogs with myxomatous mitral valve disease (MMVD) belonging to B1 stage (American College of Veterinary Internal Medicine (ACVIM) guidelines); B2, dogs with MMVD belonging to B2 stage (ACVIM guidelines); C+D, dogs with MMVD belonging to C and D stage (ACVIM guidelines); M/F, male/female; CKCS, Cavalier King Charles Spaniel; PH, pulmonary hypertension; WHWT, West Highland White Terrier.*

* *p < 0.05 in comparison with H.*

** *p < 0.001 in comparison with B1.*

*** *p < 0.001 in comparison with B2*.

Among dogs with MMVD, 39 (41%), 19 (20%), and 27 (28%) were classified as B1, B2, and C+D, respectively. Seventy-six dogs (79%) with MMVD had TR, of which 11 (14%) were considered affected by pulmonary hypertension. Among dogs with pulmonary hypertension, one (2.5%), four (21%), and six (22%) were in groups B1, B2, and C+D, respectively. Forty-three (50.5%) dogs with MMVD were receiving some cardiovascular treatment at the time of enrollment (Table 1).

### Laboratory and Cardiac Parameters

The results of laboratory analyses are shown in Table 2. The median of blood urea nitrogen (BUN) and creatinine was higher in dogs of group C+D (65 mg/dl [50–116] and 1.4 mg/dl [1.1–1.6], respectively) compared to that of dogs of group B1 (36 mg/dl [32–51] and 1.0 mg/dl [0.8–1.1], respectively; *p* < 0.001 for both comparisons). The median of BUN was also higher in dogs of group C+D compared to that of control dogs (43 mg/dl [26–50]; *p* < 0.01) and dogs of group B2 (50 mg/dl [35–58.36]; *p* = 0.04). The median serum phosphorus was higher in dogs of group C+D (3.9 mg/dl [3.4–5.1]) compared to that of dogs of group B1 (3.4 mg/dl [3.0–4.2]; *p* = 0.02). The median USG was lower in dogs of group C+D (1,014 [1,012–1,017.5]) compared to that of dogs of groups H (1,029 [1,020–1,044]; *p* < 0.01), B1 (1,026 [1,018–1,043]; *p* = 0.02), and B2 (1,028 [1,018–1,038]; *p* < 0.01).

**Table 2:** Clinical pathology parameters of 11 clinically healthy dogs (Control) and 85 dogs with myxomatous mitral valve disease grouped in different disease stages according to ACVIM guidelines.

**Variable**	**Control** ** N. = 11**	**B1** ** N. = 39**	**B2** ** N. = 19**	**C+D** ** N. = 27**	**Overall *p*-value**
BUN (mg/dl)	43 (26–50)	36 (32–51)	50 (35–58.36)	65 (50–116)^**^, ^°°^, ^#^	** <0.001**
Total protein (g/L)	72.9 (70.0–78.9)	71.8 (66.8–76.2)	69.9 (66.6–71.7)	72.5 (60.4–81.1)	0.52
Albumin (g/L)	30.7 (28.4–31.7)	31.1 (29.6–32.4)	29.4 (27.3–33.7)	28.9 (26.8–33.5)	0.13
Creatinine (mg/dl)	1.0 (1.0–1.2)	1.0 (0.8–1.1)	1.1 (0.8–1.5)	1.4 (1.1–1.6)^°°^	** <0.001**
Cholesterol (mg/dl)	272 (214–299)	236 (195–279)	210.5 (164–231)	211 (172–269)	0.10
Triglycerides (mg/dl)	74 (48–139)	62.5 (48–88)	53.5 (35–68)	69.5 (44.5–79)	0.20
Calcium (mg/dl)	10.5 (10.0–10.7)	10.2 (9.7–10.6)	10.1 (9.6–10.3)	10.3 (9.6–10.8)	0.28
Phosphorus (mg/dl)	4.0 (3.7–5.5)	3.4 (3.0–4.2)	3.6 (2.8–4.0)	3.9 (3.4–5.1)^°^	**0.02**
Na^+^ (mEq/L)	147.4 (144.2–153.6)	148 (144.5–151.3)	148.8 (143.2–150)	147.4 (144.9–152)	0.95
K^+^ (mEq/L)	4.9 (4.3-5.1)	4.6 (4.4-4.7)	4.6 (4.2-4.9)	4.3 (4.0-4.6)	0.09
USG	1,029 (1,020–1,044)	1,026 (1,018–1,043)	1,028 (1,018–1,038)	1,014 (1,012–1,017.5)^**^, ^°^, *^##^*	**0.002**
PU/CU	0.1 (0.02–0.1)	0.1 (0.1–0.3)	0.2 (0.1–0.4)	0.1 (0.1–0.3)	0.23
RBC (10^6^/μl)	7.3 (6.7–7.7)	7.0 (6.4–7.5)	6.8 (6.1–7.7)	7.3 (6.1–8.0)	0.61
WBC (10^3^/μl)	8.4 (6.0–9.9)	8.0 (7.3–9.3)	9.1 (7.3–12.6)	14.5 (9.6–22.5)^**^, ^°°^	** <0.001**
NT-proBNP (pmol/L)	372 (249–529)	434.5 (342.5–584.5)	1,423 (798–3,984) ^**^, ^°°^	4,062 (2,169–5,482)^***^, ^°°^	** <0.001**
SDMA (μmol/L)	0.4 (0.35–0.45)	0.4 (0.3–0.5)	0.4 (0.3–0.6)	0.7 (0.5–0.9)^**^, ^°°^, *^##^*	** <0.001**
ADMA (μmol/L)	1.9 (1.7–2.3)	1.8 (1.6–2.3)	2.0 (1.6–2.4)	2.5 (2.1–3.0)^*^, ^°°^	** <0.001**
L-Arg (μmol/L)	95.1 (76.4–127.3)	105.8 (85.7–126.5)	124.3 (86.6–143.8)	103 (73.1–138.5)	0.59
L-Arg/ADMA	47.2 (37.1–63.0)	50.9 (44.2–68.9)	51.9 (39.4–65.9)	41.7 (27.6–46.0)^°°^, ^#^	** <0.001**

*Data are presented as median and interquartile range.*

*Significant overall P values are reported in bold.*

*N., number of dogs; ADMA, asymmetric dimethylarginine; BUN, blood urea nitrogen; L-Arg, L-arginine; L-Arg/ADMA, L-arginine to ADMA ratio; NT-proBNP, N-terminal prohormone of brain natriuretic peptide; RBC, red blood cell; PU/CU, urinary protein to creatinine ratio; SDMA, symmetric dimethylarginine; WBC, white blood cell; USG, urine specific gravity.*

* *p < 0.05 in comparison with H.*

** *p < 0.01 in comparison with H.*

*** *p < 0.001 in comparison with H.*

° *p < 0.05 in comparison with B1.*

°° *p < 0.001 in comparison with B1.*

# *p < 0.05 in comparison with B2.*

## *p < 0.01 in comparison with B2*.

The median SDMA was higher in dogs of group C+D (0.7 μmol/L [0.5–0.9]) compared to that of dogs of groups B1 (0.4 μmol/L [0.3–0.5]; *p* < 0.001), B2 (0.4 μmol/L [0.3–0.6]; *p* < 0.01), and clinically healthy dogs (0.4 μmol/L [0.35–0.45]; *p* = 0.001). The median ADMA was higher in dogs of group C+D (2.5 μmol/L [2.1–3.0]) compared to that of dogs of group B1 (1.8 μmol/L [1.6–2.3]; *p* < 0.001) and control group (1.9 μmol/L [1.7–2.3]; *p* = 0.02), but not to that of group B2 (2.0 μmol/L [1.6–2.4]; *p* = 0.05). No difference was found for median L-arginine among dogs of the different groups (overall *p* = 0.59). On the contrary, the median L-arginine/ADMA was lower in dogs of group C+D (41.7 [27.6–46.0]) compared to that of dogs of groups B1 (50.9 [44.2–68.9]; *p* < 0.001) and B2 (51.9 [39.4–65.9]; *p* = 0.03), but not to that of the control group (47.2 [37.1–63.0]; *p* = 0.172).

The median NT-proBNP was higher in dogs of groups B2 (1423 pmol/L [798–3,984]) and C+D (4,062 pmol/L [2,169–5,482]) compared to that of dogs of control group (372 pmol/L [249–529]; *p* < 0.01 and *p* < 0.001, respectively) and group B1 (434.5 pmol/L [342.5–584.5]; *p* = 0.001 and *p* < 0.001, respectively).

According to the progressive nature of MMVD, radiographic and echocardiographic parameters of cardiac remodeling and dysfunction progressively changed among groups, according to disease severity (Supplementary Table 1).

Both SDMA and ADMA were positively correlated with BUN (*r* = 0.472 and *r* = 0.391; *p* < 0.001 for both correlations) and creatinine (*r* = 0.604 and *r* = 0.462; *p* < 0.001 for both correlations).

Symmetric dimethylarginine was also positively correlated with age (*r* = 0.426; *p* < 0.001) and NT-proBNP (*r* = 0.489; *p* < 0.001). L-arginine/ADMA was negatively correlated with NT-proBNP (*r* = −0.414; *p* < 0.001), BUN (*r* = −0.438; *p* < 0.001), and creatinine (*r* = −0.457; *p* < 0.001) (Table 3). Symmetric dimethylarginine and ADMA were also positively correlated with the echocardiographic variables LA/Ao (*r* = 0.449 and *r* = 0.424; *p* < 0.001 for both correlations), lateral MV E/E**'** (*r* = 0.410 and *r* = 0.522; *p* = 0.001 and *p* < 0.001, respectively), and septal MV E/E**'** (*r* = 0.5 and *r* = 0.478; *p* < 0.001 and *p* < 0.001, respectively) (Table 4).

**Table 3:** Spearman's ranks correlations index between symmetric dimethylarginine (SDMA), asymmetric dimethylarginine (ADMA), L-arginine, L-arginine/ADMA and demographic and clinical pathology parameters obtained from 11 control dogs and 85 dogs with myxomatous mitral valve disease at different disease stages.

**Parameter**	**SDMA**		**ADMA**		**L-arginine**		**L-arginine/ADMA**	
	***r***	***P***	***r***	***P***	***r***	***P***	***r***	***P***
Age	0.426	** <0.001**	0.300	0.002	−0.106	0.30	−0.328	0.001
BW	0.103	0.32	0.220	0.03	0.385	** <0.001**	0.142	0.17
BUN	0.472	** <0.001**	0.391	** <0.001**	0.120	0.24	−0.438	** <0.001**
Total protein	0.044	0.67	0.026	0.80	−0.046	0.65	−0.029	0.77
Albumin	−0.241	0.02	−0.123	0.23	0.106	0.30	0.238	0.02
Creatinine	0.604	** <0.001**	0.462	** <0.001**	−0.094	0.36	−0.457	** <0.001**
Cholesterol	−0.019	0.85	−0.002	0.97	−0.077	0.45	−0.076	0.46
Triglycerides	0.049	0.67	0.100	0.38	0.121	0.29	0.095	0.41
Calcium	0.120	0.24	0.152	0.14	−0.001	0.02	−0.133	0.20
Phosphorus	0.099	0.33	0.207	0.04	0.190	0.06	−0.024	0.82
Na^+^	0.155	0.14	0.173	0.10	−0.071	0.50	−0.182	0.08
K^+^	0.022	0.83	0.061	0.56	0.268	0.01	0.129	0.22
USG	−0.360	0.002	−0.392	0.001	0.001	0.98	0.333	0.005
PU/CU	−0.146	0.23	−0.084	0.49	0.083	0.49	0.071	0.56
RBC	0.068	0.50	0.146	0.15	0.128	0.21	0.037	0.72
WBC	0.281	0.005	0.199	0.05	−0.016	0.87	−0.187	0.07
NT-proBNP	0.489	** <0.001**	0.336	0.008	−0.070	0.59	−0.414	** <0.001**

*Significant overall P values are reported in bold. ADMA, asymmetric dimethylarginine; BUN, blood urea nitrogen; BW, body weight; NT-proBNP, N-terminal prohormone of brain natriuretic peptide; PU/CU, urinary protein to creatinine ratio; RBC, red blood cell; SDMA, symmetric dimethylarginine; WBC, white blood cell; USG, urine specific gravity*.

**Table 4:** Spearman's ranks correlations index between symmetric dimethylarginine (SDMA), asymmetric dimethylarginine (ADMA), L-arginine, L-arginine/ADMA and radiographic and echocardiographic parameters obtained from 11 control dogs and 85 dogs with myxomatous mitral valve disease at different diseases stages.

**Parameter**	**SDMA**		**ADMA**		**L-arginine**		**L-arginine/ADMA**	
	***r***	***P***	***r***	***P***	***r***	***P***	***r***	***P***
VHS	0.284	0.03	0.365	0.004	0.330	0.01	−0.087	0.50
LA/Ao	0.449	** <0.001**	0.424	** <0.001**	0.083	0.42	−0.294	0.004
LVIDd	0.266	0.009	0.347	** <0.001**	0.305	0.003	−0.038	0.71
LVIDd-N	0.252	0.01	0.267	0.009	0.083	0.43	−0.159	0.12
LVIDs	0.011	0.91	0.138	0.18	0.330	0.001	0.139	0.18
LVIDs-N	0.011	0.91	0.105	0.31	0.169	0.103	0.020	0.84
FS (%)	0.350	<0.001	0.250	0.02	−0.145	0.17	−0.308	0.003
MV E	0.366	<0.001	0.339	<0.001	0.035	0.73	−0.266	0.009
MV A	0.043	0.68	0.078	0.45	0.001	0.99	−0.097	0.36
MV E/A	0.244	0.02	0.213	0.04	0.010	0.92	−0.145	0.17
TV E	0.107	0.40	0.046	0.72	−0.158	0.21	−0.138	0.27
TV A	−0.058	0.66	−0.089	0.49	−0.153	0.24	−0.027	0.83
TV E/A	0.042	0.75	−0.011	0.92	−0.014	0.91	−0.040	0.76
TR Vmax	0.301	0.01	0.175	0.15	0.052	0.67	−0.048	0.69
LatMV E/E**'**	0.410	**0.001**	0.522	** <0.001**	0.263	0.05	−0.196	0.14
SepMV E/E**'**	0.500	** <0.001**	0.478	** <0.001**	0.030	0.82	−0.324	0.01
TV E/E**'**	−0.131	0.35	−0.240	0.08	−0.149	0.29	−0.023	0.87

*Significant overall P values are reported in bold. VHS, vertebral heart score; LA/Ao, left atrium to aortic root diameter ratio; LVIDd, Left ventricular internal diameter in diastole; LVIDd-N, Left ventricular internal diameter in diastole normalized for body weight; LVIDs, Left ventricular internal diameter in systole; LVIDs-N, Left ventricular internal diameter in systole normalized for body weight; FS, fractional shortening; MV E, mitral valve early diastolic velocity; MV A, mitral valve late diastolic velocity; TV E, tricuspidal valve early diastolic velocity; TV A, tricuspidal valve late diastolic velocity; TR Vmax, tricuspid regurgitation peak velocity; LatMV E**'**, peak velocity of early diastolic lateral mitral annular motion; SepMV E**'**, peak velocity of early diastolic septal mitral annular motion; TV E**'**, peak velocity of early diastolic lateral tricuspid annular motion*.

In the final multivariable analysis, considering the effect of both creatinine and LA/Ao on DMAs and the L-arginine/ADMA, ADMA was significantly and positively associated with the LA/Ao (*R*^2^ = 0.18; *p* < 0.001) and SDMA with creatinine (*R*^2^ = 0.27; *p* < 0.001) whereas L-arginine/ADMA was nearly significantly and negatively associated with creatinine (*p* = 0.001) (Table 5).

**Table 5:** Final multivariable analysis for asymmetric dimethylarginine (ADMA), symmetric dimethylarginine (SDMA) and L-arginine/ADMA as response variables and LA/Ao and creatinine as explanatory variables.

	**LA/Ao**		**Creatinine**		
**Variables**	**RCE ± SE**	***P***	**RCE ± SE**	***P***	***R^**2**^***
ADMA	0.383 ± 0.104	<0.001	0.286 ± 0.184	0.12	0.18
SDMA	0.113 ± 0.042	<0.01	0.307 ± 0.074	<0.001	0.27
L-arginine/ADMA	−5.040 ± 2.816	0.08	−15.988 ± 4.992	0.001	0.16

*Regression coefficients estimates, their standard errors and the determination coefficient of the model (R^2^) were reported.*

*ADMA, asymmetric dimethylarginine; LA/Ao, left atrium to aortic root ratio; RCE, regression coefficient estimate; SDMA, symmetric dimethylarginine; SE, standard error*.

## Discussion

This study showed an increase of plasmatic DMA's concentration associated with the more advanced stage of MMVD. Indeed, both SDMA and ADMA were higher in dogs with decompensated heart disease (group C+D) when compared to dogs in the preclinical stages of valvular disease and to dogs in the control group. On the contrary, the L-arginine/ADMA was lower in dogs with decompensated MMVD when compared to those with compensated disease. Both ADMA and SDMA were positively correlated with BUN and creatinine, and with the echocardiographic parameters, LA/Ao and both lateral and septal MV E/E**'**. Finally, in the multivariable analysis evaluating the effect of an echocardiographic (i.e., LA/Ao) and renal (i.e., creatinine) variable, ADMA was more associated with the LA/Ao than creatinine, whereas the SDMA and L-arginine/ADMA showed the opposite result. These findings are similar to those of previous studies showing an increase of ADMA in dogs with experimentally induced CHF (26) but not in animals in the preclinical stages of naturally occurring MMVD (16). However, Cunningham et al. showed that in dogs with naturally occurring CHF, ADMA did not increase if compared to healthy dogs (27). The difference between our results and this latter study is probably due to different sample size of dogs enrolled, methods of classification of MMVD severity (ACVIM guidelines vs. International Small Animal Cardiac Health Council classification), and finally, to the inclusion of either dogs with both MMVD or dilated cardiomyopathy.

It has been shown that in canine myocardial cells the enzymatic activity of dimethylarginine dimethylaminohydrolase, which partly contributes to ADMA accumulation, is reduced in the failing heart (28). Furthermore, ADMA can also be excreted through the kidneys; thus, renal damage might be responsible for ADMA accumulation (29). In humans, the “cardiorenal syndrome” is a well-known pathophysiological mechanism where decreased renal function is the consequence of reduced cardiac output (30). In dogs with decompensated MMVD, the increase of BUN is a common finding, partly due to diuretic treatment used to control clinical signs of CHF, namely prerenal azotemia. However, primary renal damage or renal dysfunction secondary to a cardiac disorder cannot be completely excluded in these animals (31–33). In humans, the presence of chronic kidney disease has been associated with increased ADMA concentration (34). The positive correlation we found between creatinine and ADMA in dogs with MMVD suggests that a reduced renal function can also have an effect on ADMA in the dog. However, results of the multivariable analysis, that evaluated the combined effect of variables of both cardiac and renal function, showed that ADMA is influenced by the LA/Ao, an index of cardiac remodeling due to MMVD, rather than by renal function. Other studies are needed to confirm if ADMA might be related to renal damage in dogs with kidney disease and if it could be predictive of a negative cardiovascular outcome, as demonstrated in humans (35).

In the present study, SDMA was higher in dogs with decompensated MMVD, whereas no difference was found in asymptomatic dogs (i.e., stages B1 and B2). This finding confirms the results of a previous prospective study in which SDMA was correlated with cardiac disease severity and with echocardiographic parameters of left atrio-ventricular dilation (14). Conversely, two other retrospective studies failed to demonstrate a correlation between SDMA and MMVD progression (36, 37). The discrepancy between our study and these latter studies can be explained by differences in study design and laboratory analytical method used to determine SDMA. Our study had a prospective design and we used high-performance liquid chromatography coupled with tandem mass spectrometry, considered a reliable diagnostic tool for measuring DMAs (38), whereas SDMA concentrations were measured through high-throughput immunoassay in previous studies (36, 37). Symmetric dimethylarginine was also positively correlated with laboratory (i.e., NT-proBNP) and echocardiographic (i.e., LA/Ao and lateral and septal MV E/E**'**) indices of cardiac wall stress, remodeling, and dysfunction. In humans, SDMA is correlated with E/E**'** in patients with chronic CHF and it has been suggested that a prolonged NOS inhibition enhanced myocardial fibrotic processes, potentially modifying myocardial function (39, 40). Previous studies suggested that NT-proBNP is more likely associated with cardiac damage rather than renal injury in both humans and dogs with chronic kidney disease (41, 42). High SDMA concentrations in our dogs with decompensated MMVD were associated with renal function, as confirmed by increased BUN, creatinine, and phosphorus, and decreased USG. However, we cannot discriminate if renal dysfunction was caused by primary renal disease, impaired cardiac function, cardiovascular treatment, or a combination of these causes. The results of this study suggest that cardiorenal syndrome, in particular cardiovascular renal disorders (43), can develop in dogs with decompensated MMVD, whereas the absence of significant increases of BUN, creatinine, and SDMA in dogs with compensated MMVD likely exclude a cardiorenal syndrome in these animals. Thus, our findings confirm that SDMA is a biomarker of renal damage, but it is also likely influenced by heart disease.

The L-arginine/ADMA was significantly lower in dogs with decompensated MMVD compared to those of group B1 and clinically healthy controls. This finding is in contrast with the results of a previous study in which no significant difference was found for L-arginine/ADMA in 43 Cavalier King Charles Spaniels with MMVD at various stages, classified according to a mitral regurgitation quantitative score (44). However, that study found decreased flow mediated dilation, a reference diagnostic method to identify endothelial dysfunction in humans (45). Different results from this study and that of Moesgaard et al. (44) are likely due to differences in number and type of enrolled dogs, various breeds vs. a unique breed, and method of MMVD classification. The L-arginine/ADMA ratio is considered an index of NO bioavailability and, in humans, it is reduced in patients with endothelial dysfunction (46). Endothelial dysfunction can develop early and become progressively severe in both humans and dogs with heart disease (46, 47), but few studies have specifically evaluated endothelial dysfunction in dogs with MMVD (44, 47). The reduced L-arginine/ADMA observed in dogs of this study confirms the presence of endothelial dysfunction in animals with decompensated MMVD. This biomarker was negatively correlated with NT-proBNP and the renal parameters, BUN and creatinine. Renal dysfunction induces an increase of ADMA concentration leading to a decrease of the L-arginine/ADMA, more evident in the advanced stages of MMVD. The association between NT-proBNP and the L-arginine/ADMA has been studied in humans with CHF and it has been correlated with impaired NO production in those patients with endothelial dysfunction (48). Based on the results of our final multivariable analysis, renal dysfunction likely has a greater influence on this parameter compared to heart disease in dogs with MMVD, but further studies are needed to confirm this hypothesis.

One limitation of this study is the smaller sample size of clinically healthy dogs compared to the other groups of dogs, but it was considered appropriate according to our preliminary statistical analysis. Moreover, this group of dogs had a lower median age compared to that of dogs in the more advanced stage of MMVD. Based on a previous study, ADMA could be influenced by breed (15). Specifically, in that study, Pointers showed increased concentrations of ADMA compared to Cairn Terriers and Cavalier King Charles Spaniels. However, it should also be noted that ADMA concentrations were evaluated exclusively in healthy dogs and only in the three aforesaid breeds. Of note, no Pointer was included in our study. Therefore, further data are needed to conclusively clarify a potential breed effect on ADMA concentration.

The presence of renal dysfunction was based on different established laboratory parameters, namely BUN, creatinine, and USG; however, evaluation of the glomerular filtration rate, the standard reference method to evaluate renal dysfunction, was not used. Finally, a cardiovascular treatment effect could not be excluded. However, cardiovascular drugs used for CHF treatment (i.e., diuretics, ACE-I, and nitrovasodilators) did not show any influence on ADMA concentrations in humans (49).

In dogs, diuretic therapy has been shown to impair renal function, leading to an increase of renal parameters. Two studies obtained contrasting results regarding the effect of therapies on SDMA concentrations in dogs with MMVD (36, 37). In the study of Savarese et al. no therapies effect has been shown (36), whereas in the other study increasing doses of furosemide seemed to be associated with an increase of SDMA concentrations. However, the combined analysis of therapy effect with MMVD ACVIM stage did not show any significant result (37). Cardiovascular therapy effect on SDMA in dogs with MMVD is still unclear and, so far, it is not possible to discriminate if the variation of SDMA concentrations is more influenced by cardiovascular drugs effect on glomerular filtration rate, or by the cardiac disease itself. Furthermore, some dogs were referred cases and, occasionally, they were already receiving several different cardiovascular treatments from the referring veterinarian that were not always in agreement with the ACVIM guidelines. Thus, further investigations considering a more standardized dog's population are needed. Finally, the dogs included in the study did not receive a standardized diet, thus an effect of the diet composition on the plasmatic concentration of DMAs cannot be excluded.

## Conclusion

The results of this study showed an increase of ADMA and SDMA and a reduction of the L-arginine/ADMA in dogs in the more advanced stage of MMVD. Asymmetric dimethylarginine can be considered a biomarker of cardiac and endothelial dysfunction in dogs with decompensated MMVD. However, an impaired renal function might contribute for its increase in these animals. At the same time, SDMA is mainly dependent on renal function but the presence of advanced valvular disease can also influence its concentration.

## Data Availability Statement

The raw data supporting the conclusions of this article will be made available by the authors, without undue reservation.

## Ethics Statement

The animal study was reviewed and approved by the University of Padua Animal Welfare Ethics Committee (authorization number 26/2017). Written informed consent was obtained from the owners for the participation of their animals in this study.

## Author Contributions

CG and HP: Conceptualization. BC: formal analysis. CV, CG, MBT, GR, CA, LB, and HP: investigation and data collection. CV: writing-original draft preparation. CV, CG, MBT, GR, CA, LB, BC, and HP: writing-review and editing. All authors contributed to the article and approved the submitted version.

## Funding

This work was supported by a grant of University of Padua, Italy, to Dr. Poser (SID Year: 2017- Protocol number: BIRD173881).

## Conflict of Interest

The authors declare that the research was conducted in the absence of any commercial or financial relationships that could be construed as a potential conflict of interest.

## Publisher's Note

All claims expressed in this article are solely those of the authors and do not necessarily represent those of their affiliated organizations, or those of the publisher, the editors and the reviewers. Any product that may be evaluated in this article, or claim that may be made by its manufacturer, is not guaranteed or endorsed by the publisher.
