# Supplementary material for: Plasmatic Dimethylarginines in Dogs With Myxomatous Mitral Valve Disease
Source: Front Vet Sci. 2021 Sep 16;8:738898. doi: 10.3389/fvets.2021.738898 (PMC8481685; doi:10.3389/fvets.2021.738898)
Supplement: Supplementary file 1 [file Table_1.DOCX]

**Supplementary table 1**. Radiographic and echocardiographic parameters of 11 clinically healthy dogs (H) and 85 dogs with myxomatous mitral valve disease, grouped according to American College of Veterinary Internal Medicine (ACVIM) guidelines.

| **Variable** | **Control (H)**  **N = 11** | **B1**  **N = 39** | **B2**  **N = 19** | **C+D**  **N = 27** | **Overall p-value** |
| --- | --- | --- | --- | --- | --- |
| **VHS** | 10.6 (10-10.8) | 10.8 (10.5-11.2) | 11.8 (11.3-12.7) ^**,°°^ | 12.8 (11.3-13.5) ^**,°°^ | **< 0.001** |
| **LA/Ao** | 1.4 (1.3-1.5) | 1.5 (1.4-1.6) | 2.1 (1.9-2.6) ^***,°°°^ | 2.3 (2.2-3.0) ^***,°°°^ | **< 0.001** |
| **LVIDd (cm)** | 2.7 (1.8-3.1) | 3.0 (2.8-3.4) | 3.6 (3.1-4.2) ^*,°°^ | 3.9 (3.5-4.4) ^*,°°^ | **< 0.001** |
| **LVIDd-N** | 1.4 (1.2-1.5) | 1.5 (1.4-1.6) * | 1.9 (1.8-2.2) ^**,°°°^ | 2.0 (1.9-2.2)^*,°°°^ | **< 0.001** |
| **LVIDs (cm)** | 1.5 (1.2–2.0) | 1.9 (1.6-2.2) | 2.1 (1.6-2.4) | 1.9 (1.6-2.7) | 0.37 |
| **LVIDs-N** | 0.8 (0.7-1.0) | 0.9 (0.8-1.0) | 1.1 (0.1-1.2) ^*,°°^ | 1.1 (0.8-1.2) | **0.01** |
| **FS%** | 41.9 (34.9-42.7) | 36.2 (30-41.4) | 44.8 (40.7-48.3) ^°°^ | 51.1 (40-57.2)^°°°^ | **< 0.001** |
| **MV E (cm/s)** | 66.1 (62.6-81.1) | 73.5 (61.7-84.2) | 138 (107-150.3) ^***,°°°^ | 156.7 (133.3-167.3)^***, °°°^ | **< 0.001** |
| **MV A (cm/s)** | 60.8 (45.8-69.36) | 65.5 (56.3-83) | 75 (65.3-95.2) | 64.5 (55.1-78.9) | 0.08 |
| **MV E/A** | 1.2 (0.9-1.4) | 1.1 (0.9-1.3) | 1.7 (1.3-2.3) °°° | 2.1 (1.7-2.7) ^*,°°°^ | **< 0.001** |
| **TV E (cm/s)** | 44.6 (40.2-46.4) | 50.1 (43.1-58.6) | 61.6 (43.7–72.4) | 57.4 (42.3-75.3) | 0.21 |
| **TV A (cm/s)** | 44.1 (30.1-54.1) | 45.4 (37–52.3) | 44.0 (32.8-62.3) | 41.4 (33.3-59.1) | 0.78 |
| **TV E/A** | 1.2 (0.8-1.5) | 1.2 (0.9-1.3) | 1.4 (1.0-1.6) | 1.3 (1.0-1.5) | 0.47 |
| **TR Vmax (m/s)** | NA | 2.5 (2.1-2.7) | 3.1 (2.6–3.1) | 2.9 (2.6-3.4)° | **0.02** |
| **LatMV E/E´** | 7.6 (6.5-9.4) | 8.4 (6.8-9.3) | 9.0 (7.4-11.9) | 12.1 (10.4-13.1)^*,°^ | **0.01** |
| **SepMV E/E´** | 8.5 (7.8-12.0) | 10.0 (8.8-11.2) | 10.4 (7.8–11.4) | 12.7 (10.3–14.9) | 0.08 |
| **TV E/E´** | 5.3 (4.1-5.7) | 5.1 (4.2-6.2) | 3.5 (3.3–3.9) ^*,°^ | 4.3 (3.1-5.6) | **0.01** |

Data are presented as median and interquartile range. Significant Overall p-values are reported in bold.

N, number of dogs; VHS, vertebral heart score; LA/Ao, left atrial to aortic root diameter ratio; LVIDd, Left ventricular internal diameter in diastole; LVIDd-N, Left ventricular internal diameter in diastole normalized for body weight; LVIDs, Left ventricular internal diameter in systole; LVIDs-N, Left ventricular internal diameter in systole normalized for body weight; FS, fractional shortening; MV E, mitral valve early diastolic velocity; MV A, mitral valve late diastolic velocity; TV E, tricuspid valve early diastolic velocity; TV A, tricuspid valve late diastolic velocity; TR Vmax, tricuspid regurgitation peak velocity; NA, not available; LatMV E**´**, peak velocity of early diastolic lateral mitral annular motion; SepMV E**´**, peak velocity of early diastolic septal mitral annular motion; TV E**´**, peak velocity of early diastolic lateral tricuspid annular motion.

^*^ P < 0.05 in comparison with H

^**^P < 0.01 in comparison with H

^***^ P < 0.001 in comparison with H

^°^ P < 0.05 in comparison with B1

^°°^ P < 0.01 in comparison with B1

^°°°^ P < 0.001 in comparison with B1
